# Systematic lymph node sampling at operation results in more node positive staging in NSCLC

**DOI:** 10.1186/1749-8090-10-S1-A346

**Published:** 2015-12-16

**Authors:** J Whiteley, G Beattie, A McKay, A Kirk, M Asif

**Affiliations:** 1 Golden Jubilee National Hospital, Clydebank, Dunbartonshire G81 4DY, UK

## Background/Introduction

It has been shown that the survival of patients with NSCLC is related to nodal involvement. Both BTS and SIGN guidelines recognise that simple nodal sampling is not adequate to accurately stage NSCLC and recommend at least systematic sampling of mediastinal nodes. In the West of Scotland all patients with node positive disease are considered for adjuvant chemotherapy. This requires accurate staging of lymph nodes.

## Aims/Objectives

We report on the nodal staging of all patients in 2012 and 2013 who underwent lung resection.

## Method

All patients who underwent surgery for NSCLC at The Golden Jubilee National Hospital in 2012 and 2013 were included. Data were collected on histological diagnosis, T and N staging from histology reports, the lymph nodes stations sampled at operation and the operating surgeon. Adequate lymph node sampling was considered to have been performed if more than three mediastinal lymph node stations were sampled, as this is a quality performance indicator set out by NHS Scotland.

## Results

583 patients underwent lung resection. There was no difference in the proportion of patients with nodal disease between operating consultant (p = 0.120). In this cohort, as the number of lymph node stations sampled increased, the proportion of node positive disease increased from 16% and 19% if only 1 or 2 lymph node stations were sampled respectively, to 42% node positive if more than 6 stations were sampled (p = 0.002). When 3 or more mediastinal lymph node stations were sampled, then the detection of nodal disease increased by 50% (p = 0.006).

## Discussion/Conclusion

By sampling more mediastinal lymph nodes, more diseased lymph nodes were detected. This meant these patients were more accurately staged and considered for adjuvant chemotherapy, therefore potentially increasing their chances of survival.

